# *BARD1* deletion in a patient with suspected hereditary colorectal cancer

**DOI:** 10.1038/s41439-024-00267-y

**Published:** 2024-03-15

**Authors:** Nobue Takaiso, Issei Imoto, Akiyo Yoshimura, Akira Ouchi, Koji Komori, Hiroji Iwata, Yasuhiro Shimizu

**Affiliations:** 1 Risk Assessment Unit, Aichi Cancer Center Hospital, Nagoya, Japan; 2 Aichi Cancer Center Research Institute, Nagoya, Japan; 3 Department of Breast Oncology, Aichi Cancer Center Hospital, Nagoya, Japan; 4 Department of Gastroenterological Surgery, Aichi Cancer Center Hospital, Nagoya, Japan

**Keywords:** Genetic testing, Cancer genetics

## Abstract

Deleterious germline variants in the *BRCA1-associated ring domain* (*BARD1*) gene moderately elevate breast cancer risk; however, their potential association with other neoplasms remains unclear. Here, we present the case of a 43-year-old female patient diagnosed with sigmoid colon adenocarcinoma whose maternal family members met the Amsterdam Criteria II for Lynch syndrome. Comprehensive multigene panel testing revealed a heterozygous *BARD1* exon 3 deletion.

The full-length BRCA1-associated ring domain 1 (BARD1) protein contains an N-terminal RING domain and C-terminal tandem BRCT repeats, similar to BRCA1^1^. BARD1 and BRCA1 form a heterodimer through the interaction of their RING-finger domains; this heterodimer plays crucial roles in several tumor-suppressive functions related to DNA repair and apoptosis. *BARD1* has emerged as a moderate-risk gene for hereditary breast cancer (BC), particularly triple-negative BC (TNBC)^2,3^. Pathogenic/likely pathogenic (P/LP) *BARD1* variant carriers have a 17–30% lifetime risk of BC, prompting the National Comprehensive Cancer Network (NCCN) guidelines to recommend annual mammograms starting at age 40, along with annual breast magnetic resonance imaging (MRI)^4^. The recommended timing of MRI for P/LP *BARD1* variant carriers depends on additional risk factors, including age, family history, breast density, and patient preference. Risk-reducing mastectomy is not recommended for P/LP *BARD1* variant carriers but may be considered depending on family history^4^. However, the association between P/LP *BARD1* variants and an increased risk of other cancers, including colorectal cancer (CRC), remains unclear. Despite the increasing number of P/LP *BARD1* variants identified in families with CRC aggregation, the data are limited, and no definitive associations have been established^5,6^. Recently, a germline heterozygous deletion of *BARD1* exons 8–11 was reported in a family diagnosed with familial colorectal cancer type X syndrome, meeting the Revised Amsterdam Criteria (Amsterdam Criteria II) for Lynch syndrome (LS) without any P/LP variants in the DNA mismatch repair (MMR) genes^7^.

We present the case of a 42-year-old Japanese female (III-4; Fig. 1) who underwent a medical assessment following the detection of fecal occult blood during a medical check-up. Subsequent colonoscopy revealed a tumor in the sigmoid colon. The pathological diagnosis of the biopsy specimen was compatible with colon adenocarcinoma. She was referred to our hospital for a colonoscopy and contrast-enhanced whole-body computed tomography scan, which led to a diagnosis of sigmoid colon cancer (cT2N1aM0; TNM clinical staging, cStage IIIa)^8^. The patient underwent laparoscopic high anterior resection, and pathological examination revealed a well-differentiated adenocarcinoma (tub1 > 2; pT2N0M0; TNM pathological staging, pStage I)^8^. Universal MMR deficiency (dMMR) screening by immunohistochemistry (IHC) for MMR and BRAF V600E proteins in resected tumor samples revealed significant downregulation of the MLH1 and PMS2 proteins, indicating dMMR, while BRAF V600E was negative. Notably, the proband had a family history of CRC (Fig. 1A). Her grandmother (I-4, deceased in her 70s) and mother (II-12, diagnosed in her 40s and again at age 65) were both affected by CRC. As a result, this family fulfilled the Amsterdam Criteria II for LS^9^. Contrast-enhanced MRI revealed a small, lobulated mass in the right breast of the proband, but histopathological examination of the biopsy specimens revealed no evidence of proliferative changes.

**Fig. 1:**
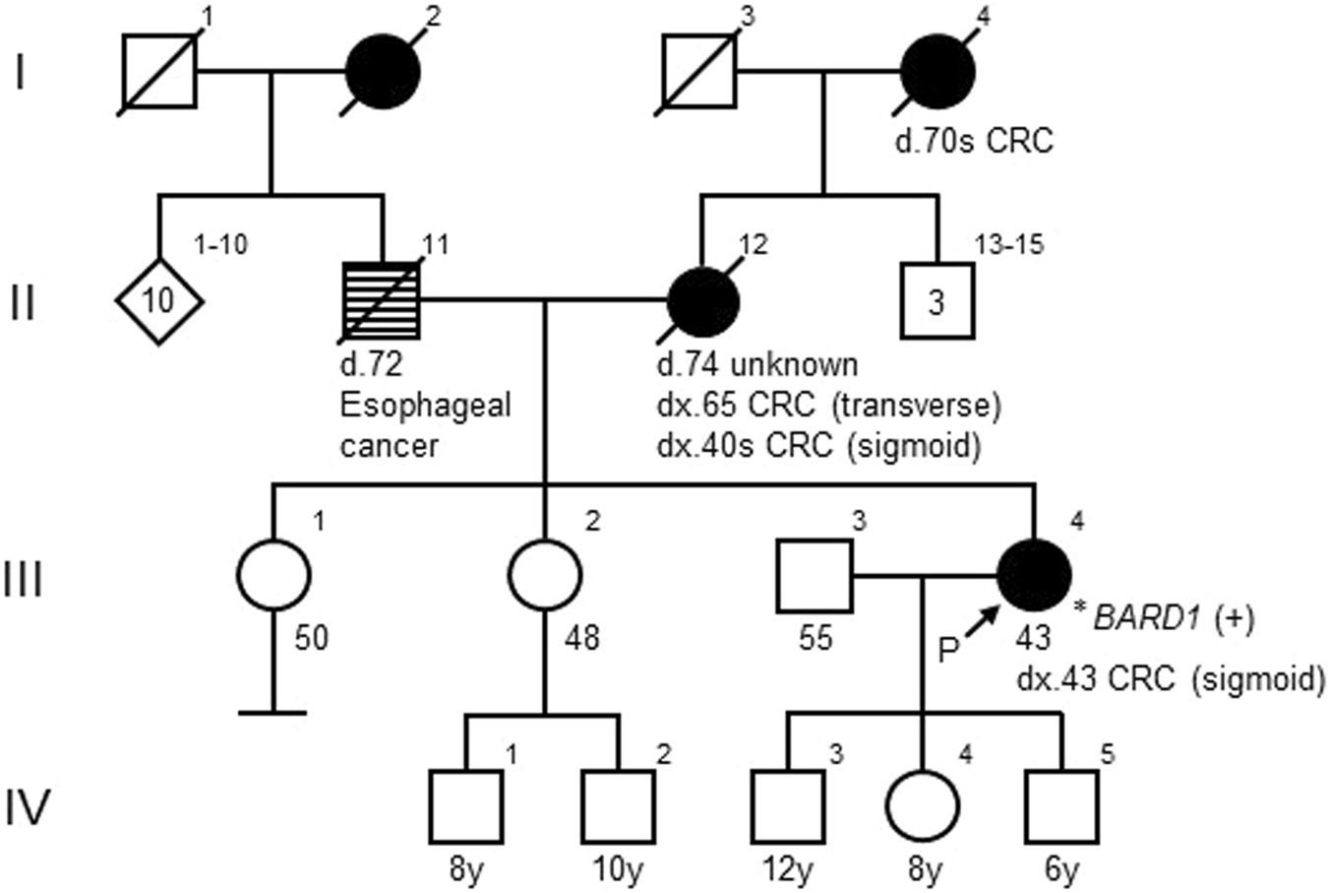
Family pedigree. The arrow indicates the proband (P). The filled symbols indicate individuals affected by colorectal cancer (CRC).

The patient was referred to our clinical genetics department for hereditary tumor risk assessment because her personal and family history met the Amsterdam Criteria II for LS, and the dMMR screening results were positive. A complete physical examination revealed no phenotypic features suggestive of any specific syndrome. For a definitive diagnosis of hereditary CRC, we proposed several commercially available genetic tests, including multigene panel testing (MGPT). Following pretest genetic counseling and obtaining informed consent, the patient opted for and underwent MGPT with an 84-gene hereditary cancer panel via next-generation sequencing (NGS; Invitae Multi-Cancer Panel, https://www.invitae.com/en/providers/test-catalog/test-01101) to evaluate as many CRC predisposition genes as possible. Pretest genetic counseling primarily addressed the clinically suspected genes while also discussing the potential incidental findings within the panel. The Invitae 84-gene panel was generated using Illumina NGS technology. The genes were targeted and sequenced via a custom short-read NGS assay in which genomic DNA was extracted from blood with beads designed to capture exons, 20 bases of the flanking introns, and certain noncoding regions as previously described^10^. A bioinformatics pipeline was used to align the sequencing reads, and community standard and custom algorithms were used to identify single-nucleotide variants, small insertions or deletions (indels), large indels, structural variants, and exon-level copy number variants (CNVs)^10–12^. The identified variants were interpreted using Sherloc^13^, a proprietary, point-based framework based on the joint consensus guidelines from the American College of Medical Genetics and Genomics and the Association for Molecular Pathology (ACMG/AMP)^14^.

No P/LP variants in established CRC susceptibility genes, including MMR genes, were detected in the proband, suggesting that the MLH1 protein expression found in our universal dMMR screening was downregulated by acquired epigenetic mechanisms such as *MLH1* promoter hypermethylation. An unexpected heterozygous out-of-frame deletion of *BARD1* exon 3 (NM_000465.4) was initially detected in the 84-gene MGPT (Fig. 2A) and subsequently confirmed using exon-focused array comparative genomic hybridization (aCGH). To our knowledge, this CNV of *BARD1*, NC_000002.12 (NM_000465.4):c.(215 + 1_216-1)_(364 + 1_347-1)del, has never been reported in disease-related databases, including the Human Gene Mutation Database (HGMD, https://my.qiagendigitalinsights.com/bbp/view/hgmd/pro/start.php); ClinVar (https://www.ncbi.nlm.nih.gov/clinvar/); or population databases, including 8.3KJPN-SV (https://jmorp.megabank.tohoku.ac.jp/) and gnomAD SVs v2.1 (https://gnomad.broadinstitute.org/). This CNV is predicted to generate an out-of-frame transcript, possibly leading to a premature termination codon and causing a loss of function event, so-called nonsense-mediated mRNA decay (NMD). The deletion of exon 3 also causes the deletion of the RING domain of the protein, which is essential for BRCA1-BARD1 heterodimer formation and E3 ubiquitin ligase activity, as well as for the stability of BRCA1 (Fig. 2B). Therefore, the functional consequences of this variant are expected to be severe, even if NMD is not caused. Segregation analysis could not be performed for all affected and unaffected relatives in this case. According to the ACMG/AMP criteria^14^, this CNV was classified as LP (PVS1 and PM2). Although *BARD1* deletions have been reported in patients diagnosed with various cancers, including CRC (Supplementary Table S1), the germline deletion of *BARD1* exon 3 represents a novel, previously unreported pathogenic variant.

**Fig. 2:**
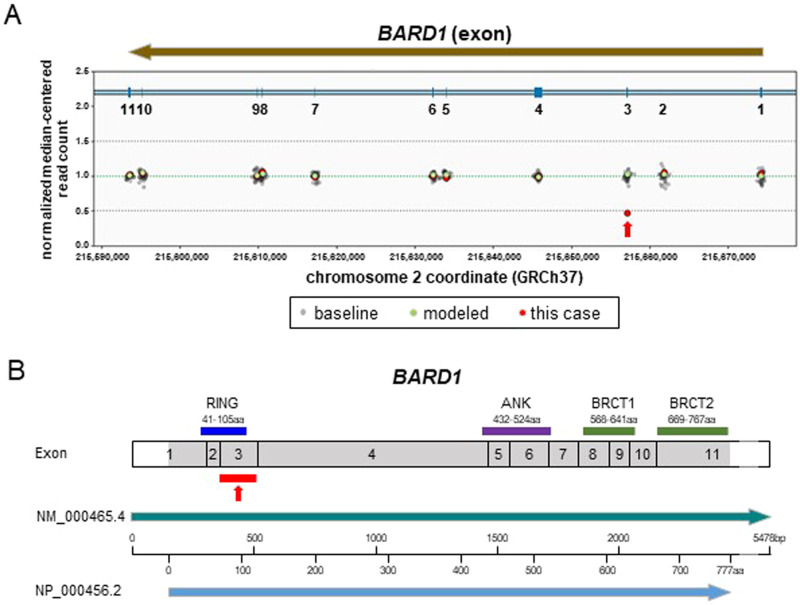
The deleted exon and structure of *BARD1*. **A** The results of the copy number analysis of *BARD1* detected by the 84-gene MGPT. The red arrow indicates the deletion of *BARD1* exon 3. **B** Structure of *BARD1*. Exon structure, coding exons (gray area), and domain composition of full-length BARD1 based on NM_000465.4 (green arrow) and NP_000456.2 (red arrow), with scales at the nucleotide (bp) and amino acid (aa) levels. BARD1 has an N-terminal RING-finger domain (RING), three centrally located ankyrin repeats (ANK), and two C-terminal BRCT (BRCA1 C-terminus) domains (BRCT1 and BRCT2).

While the full-length BARD1 protein participates in both BRCA1-dependent and BRCA1-independent tumor-suppressive pathways, multiple exon-skipping BARD1 isoforms, many of which have agonistic cancer susceptibility potential, have been reported^15^. To date, ~19 BARD1 isoforms have been identified, all of which are expressed in colon and CRC tissues^15,16^. Since some isoforms lack exon 3, the allele lacking exon 3 may express isoforms without specific exons, including exon 3, instead of causing NMD. Notably, several BARD1 isoforms lacking exon 3, such as BARD1β (lacking exons 2 and 3) and BARD1δ (lacking exons 2–6), antagonize full-length BARD1 and confer cancer susceptibility and oncogenicity^15,17^. Consequently, in the present study, the allele lacking full-length BARD1 expression due to the exon 3 deletion was likely to cause the loss or antagonism of tumor-suppressive functions, even in the presence of expressed isoforms. Because somatic pathogenic variants occurring as a second hit in the intact *BARD1* allele, homologous recombination deficiency status, and the mRNA-level expression status of each isoform from each *BARD1* allele were not analyzed in the tumor tissues from this patient, it is unclear whether the detected germline *BARD1* variant contributed to the pathogenesis of CRC or was just an incidental alteration; if the former is true, how this variant may cause CRC is also unknown. Additional research-based analyses of tumors would be useful to clarify the involvement of BARD1 dysfunction in the pathogenesis of CRC in the present case.

Only a few CRC cases with germline *BARD1* deleterious variants have been reported^6,7,18,19^. Of the four reported CRC cases, three had variants predicted to cause exon skipping: exon 3 deletion (this case), exons 8–11 deletion^7^, and NM_000465.3:c.1811-2A>G^6^. Although these three variants were observed in families with CRC aggregation, only two patients, including this patient, with gross *BARD1* deletions met the Amsterdam Criteria II for LS^7^. Larger population-based studies are needed to validate the potential association between pathogenic *BARD1* variants and elevated CRC risk^7^. Furthermore, future studies demonstrating the cosegregation of gross *BARD1* deletions with the phenotype in large, affected families would be valuable in establishing an unequivocal causal link between this type of *BARD1* variant and CRC aggregation. Currently, it is not feasible to assess CRC risk in at-risk family members based solely on the presence or absence of the *BARD1* exonic deletion. However, if a causal relationship is established in the future, CRC risk could be estimated according to the deletion status of this gene.

## HGV database

The relevant data from this Data Report are hosted at the Human Genome Variation Database at 10.6084/m9.figshare.hgv.3371.

## Supplementary information

Supplementary data (Supplementary Table S1 and Supplementary References)
