# Supplementary material for: BARD1 deletion in a patient with suspected hereditary colorectal cancer
Source: Hum Genome Var. 2024 Mar 15;11:11. doi: 10.1038/s41439-024-00267-y (PMC10940602; doi:10.1038/s41439-024-00267-y)
Supplement: Supplementary file 1 — Supplementary data (Supplementary Table S1 and Supplementary References) [file 41439_2024_267_MOESM1_ESM.docx]

**Supplementary data**

**Supplementary Table S1**

**Reported germline gross deletions of the *BARD1* gene**

|  | *BARD1* exon(s) involved | Reported phenotype* | Reference |
| --- | --- | --- | --- |
| 1 | Exon 3 | Colon cancer | Present case |
| 2 | Entire gene | Breast cancer | S1 |
| 3 | Entire gene | Ovarian cancer | S2 |
| 4 | Exons 1–11 | Breast cancer | S3 |
| 5 | Exons 1–11 | Wilms tumor | S4 |
| 6 | Exon 1 | Breast cancer | S5 |
| 7 | Exon 2 | Breast cancer | S6 |
| 8 | Exons 2–11 | Breast cancer | S7 |
| 9 | Exons 5–6 | Rectal cancer | 12 |
| 10 | Exons 5–7 | Breast cancer | S8 |
| 11 | Exons 7–8 | Ovarian cancer | S9 |
| 12 | Exons 7–11 | Breast cancer | S8 |
| 13 | Exons 8–11 | Ovarian cancer | S2 |
| 14 | Exons 8–11 | Rectal cancer | 7 |
| 15 | Exon 9 | Breast cancer | S7 |
| 16 | Exon 9 | Renal cancer | S10 |
| 17 | Exons 10–11 | Breast cancer | S7 |

*Reported phenotype of the index case (proband).

**Supplementary References**

S1. Sabatier R, Adélaïde J, Finetti P, Ferrari A, Huiart L, Sobol H, Chaffanet M, Birnbaum D, Bertucci F. BARD1 homozygous deletion, a possible alternative to BRCA1 mutation in basal breast cancer. Genes Chromosomes Cancer. 2010;49:1143–1151.

S2. Carter NJ, Marshall ML, Susswein LR, Zorn KK, Hiraki S, Arvai KJ, Torene RI, McGill AK, Yackowski L, Murphy PD, Xu Z, Solomon BD, Klein RT, Hruska KS. Germline pathogenic variants identified in women with ovarian tumors. Gynecol Oncol. 2018;151:481–488.

S3. Kwong A, Shin VY, Chen J, Cheuk IWY, Ho CYS, Au CH, Chan KKL, Ngan HYS, Chan TL, Ford JM, Ma ESK. Germline mutation in 1338 BRCA-negative Chinese hereditary breast and/or ovarian cancer patients: Clinical testing with a multigene test panel. J Mol Diagn. 2020;22:544–554.

S4. Byrjalsen A, Hansen TVO, Stoltze UK, Mehrjouy MM, Barnkob NM, Hjalgrim LL, Mathiasen R, Lautrup CK, Gregersen PA, Hasle H, Wehner PS, Tuckuviene R, Sackett PW, Laspiur AO, Rossing M, Marvig RL, Tommerup N, Olsen TE, Scheie D, Gupta R, Gerdes AM, Schmiegelow K, Wadt K. Nationwide germline whole genome sequencing of 198 consecutive pediatric cancer patients reveals a high incidence of cancer prone syndromes. PLoS Genet. 2020;16:e1009231.

S5. Tung N, Battelli C, Allen B, Kaldate R, Bhatnagar S, Bowles K, Timms K, Garber JE, Herold C, Ellisen L, Krejdovsky J, DeLeonardis K, Sedgwick K, Soltis K, Roa B, Wenstrup RJ, Hartman AR. Frequency of mutations in individuals with breast cancer referred for BRCA1 and BRCA2 testing using next-generation sequencing with a 25-gene panel. Cancer. 2015;121:25–33

S6. Adedokun B, Zheng Y, Ndom P, Gakwaya A, Makumbi T, Zhou AY, Yoshimatsu TF, Rodriguez A, Madduri RK, Foster IT, Sallam A, Olopade OI, Huo D. Prevalence of inherited mutations in breast cancer predisposition genes among women in Uganda and Cameroon. Cancer Epidemiol Biomarkers Prev. 2020;29:359–367.

S7. Benito-Sánchez B, Barroso A, Fernández V, Mercadillo F, Núñez-Torres R, Pita G, Pombo L, Morales-Chamorro R, Cano-Cano JM, Urioste M, González-Neira A, Osorio A. Apparent regional differences in the spectrum of BARD1 pathogenic variants in Spanish population and importance of copy number variants. Sci Rep. 2022;12:8547.

S8. Kaneyasu T, Mori S, Yamauchi H, Ohsumi S, Ohno S, Aoki D, Baba S, Kawano J, Miki Y, Matsumoto N, Nagasaki M, Yoshida R, Akashi-Tanaka S, Iwase T, Kitagawa D, Masuda K, Hirasawa A, Arai M, Takei J, Ide Y, Gotoh O, Yaguchi N, Nishi M, Kaneko K, Matsuyama Y, Okawa M, Suzuki M, Nezu A, Yokoyama S, Amino S, Inuzuka M, Noda T, Nakamura S. Prevalence of disease-causing genes in Japanese patients with BRCA1/2-wildtype hereditary breast and ovarian cancer syndrome. NPJ Breast Cancer. 2020;6:25.

S9. Rofes P, Del Valle J, Torres-Esquius S, Feliubadaló L, Stradella A, Moreno-Cabrera JM, López-Doriga A, Munté E, De Cid R, Campos O, Cuesta R, Teulé Á, Grau È, Sanz J, Capellá G, Díez O, Brunet J, Balmaña J, Lázaro C. BARD1 pathogenic variants are associated with triple-negative breast cancer in a Spanish Hereditary Breast and Ovarian Cancer Cohort. Genes (Basel). 2021;12:150.

S10. Hartman TR, Demidova EV, Lesh RW, Hoang L, Richardson M, Forman A, Kessler L, Speare V, Golemis EA, Hall MJ, Daly MB, Arora S. Prevalence of pathogenic variants in DNA damage response and repair genes in patients undergoing cancer risk assessment and reporting a personal history of early-onset renal cancer. Sci Rep. 2020;10:13518.
